# Valorization of Strawberry Juice Production Wastewater: Possibilities for Polyphenols Recovery and Plant Biostimulant Production

**DOI:** 10.3390/foods13203224

**Published:** 2024-10-10

**Authors:** Ivana Danilov, Vanja Vlajkov, Zdravko Šumić, Anita Milić, Aleksandra Tepić Horecki, Tatjana Dujković, Nemanja Živanović, Nataša Simin, Marija Lesjak, Jovana Grahovac

**Affiliations:** 1 Faculty of Technology Novi Sad, University of Novi Sad, Bulevar cara Lazara 1, 21000 Novi Sad, Serbia; ivana.pajcin@uns.ac.rs (I.D.); sumic@uns.ac.rs (Z.Š.); anitavakula@uns.ac.rs (A.M.); tepical@uns.ac.rs (A.T.H.); tatjana.dujkovic@uns.ac.rs (T.D.); johana@uns.ac.rs (J.G.); 2 Faculty of Sciences, University of Novi Sad, Trg Dositeja Obradovića 3, 21000 Novi Sad, Serbia; nemanja.zivanovic@dh.uns.ac.rs (N.Ž.); natasa.simin@dh.uns.ac.rs (N.S.); marija.lesjak@dh.uns.ac.rs (M.L.)

**Keywords:** *Bacillus* sp., bioreactor, circular bioeconomy, bioprocess solution, waste valorization, antioxidant activity, fruit processing, plant biostimulant, plant growth promotion, tomato

## Abstract

Fruit juice production is one of the most important branches of the food and beverage industry, considering both the market size and demand. It is also one of the largest generators of industrial wastewater, considering the large consumption of fresh water during fruit processing. Hence, the appropriate treatment strategies are of the utmost importance to minimize the environmental footprint of food industry effluents. This study aimed to investigate the valorization routes for strawberry juice production wastewater (SJPW), both in terms of nutrient recovery and a circular approach to its utilization as a medium for plant biostimulant production. The results show a low antioxidant capacity and low content of polyphenols in SJPW; however, promising results were obtained for the in vitro seed germination and tomato growth promotion when investigating a biostimulant based on *Bacillus* sp. BioSol021, which was cultivated using SJPW in a lab-scale bioreactor, with root and shoot length improvements of approximately 30% and 25%, respectively, compared to the control samples. The plant growth promotion (PGP) traits indicated the ability of IAA production, in a concentration of 8.55 ± 0.05 mg/L, and the enzymatic activity was evaluated as through the enzymatic activity index (EAI), achieving the following: 2.26 ± 0.04 for cellulolytic activity, 2.49 ± 0.08 for hemicellulolytic activity, 2.91 ± 0.16 for pectinolytic activity, and 1.05 ± 0.00 for proteolytic activity. This study opens a new chapter of possibilities for the development of techno-economically viable circular bioprocess solutions aimed at obtaining value-added microbial products for sustainable agriculture based on the valorization of food industry effluents thus contributing to more sustainable food production at both the agricultural and industrial levels.

## 1. Introduction

Fruit juice production is a well-known industry that generates a large volume of wastewater through the different stages of processing, including washing, soaking, blanching, packing, and extraction. The aforementioned processes require plenty of fresh water, consequently leading to the generation of excessive amounts of wastewater, and the estimations imply that the production of 1 L of juice generates nearly 10–22.5 L of wastewater [1]. Taking into account the content of the generated wastewater characterized by high chemical oxygen demand (COD), suspended solids, dissolved solids, low-nutrient (nitrogen and phosphorous) concentrations, and low pH, it is clear that its discharge without proper treatment is concerning from the ecological point of view [2]. The juice industry wastewater has to be properly treated to achieve the necessary quality level that complies with the regulations defining discharge into surface waters, regarding pH value and the residual compounds content, with an emphasis on their organic load [3]. The fruit juice market size was estimated at US$ 153.8 billion in 2023, with an expected CAGR (compound annual growth rate) of 3.8% to achieve US$ 216.6 billion by 2032 [4]. The future of the fruit juice industry is, among others, defined through sustainable development aspects, with the aim to achieve social development and environmental preservation [5]. On the other hand, fruit juice production effluents are valuable resources in terms of residual nutrients content such as flavonoids, polyphenols, pigments, mono- and polysaccharides, dietary fibers, and proteins [1]. Thus, novel routes for their simultaneous treatment and valorization have been investigated and developed to prevent nutrient loss and the related ecological risks and hazards of their disposal, with a particular focus on biotreatments providing circular economy-based zero waste solutions [1,2,3,5].

Fresh strawberries contain over 90% water, as well as around 4.9 g/100 g FW of sugars, 0.67 g/100 g FW of proteins, and 2 g/100 g FW of fibers [6]. All the aforementioned nutritional parameters strongly depend on the strawberry variety and growing conditions, thus sugar content in the Clery variety was found to range from 5.4 to 6.8 g/100 g in the study by Simkova et al. (2023) [7]. As for the Clery variety strawberries grown in Serbia, glucose and fructose content were found to be in the range of 2.22 ± 0.10 g/100 g and 2.50 ± 0.24 g/100 g, respectively [8].

During fruit processing, solid waste is mostly investigated as the potential source of value-added bioactive compounds, such as phenolic acids, flavonoids, coumarins and lignans [9,10,11], with a wide range of possible applications in the pharmaceutical, food, and cosmetic industries [12]. However, due to the solubility of antioxidant compounds, a large share of their total amount also eventually ends up in liquid waste streams after fruit processing [13]. The abundance of fruit processing wastewater due to large water consumption in the fruit processing industry makes it a viable source of circular bioactive compounds. Although antioxidant compounds are usually present in small concentrations in fruit processing wastewater, many separation, purification, and concentration techniques have been investigated and developed so far for their recovery, including physico-chemical processes (adsorption, solvent extraction, electrochemical oxidation), as well as pressure-driven and membrane-based processes (ultrafiltration, nanofiltration, liquid membrane technology) [12,14,15].

Furthermore, fruit-based waste contains a number of free sugars, mostly glucose, fructose, and sucrose, which can be used as excellent carbon sources for the cultivation of microorganisms, complemented by other nitrogen and phosphorus sources as well. Strawberry juice wastewater contains approximately 13 g/L of sucrose, 20 g/L of glucose, and 23 g/L of fructose, depending on the fruit juice processing technology. Taking this into account, they represent a suitable feedstock that can be efficiently exploited for biotechnological production, i.e., the growth and metabolic activity of beneficial microbial active components [16]. The ideology of a waste biorefinery is based on the efficient use of available resources and consequent reduction in environmental footprint [17]. Therefore, it can be explained that the concepts of circular economy and bioeconomy are convergent, creating a new term ‘circular bioeconomy’, which relies on the conversion of waste materials into the wide palette of value-added bio-based products [17,18,19].

One of the promising strategies for facing the global problem of food scarcity lies in incorporating biostimulants in agricultural production practice, as an eco-friendly solution that aligns with the United Nations’ sustainable development goals (SDGs). Plant biostimulants represent microorganisms or compounds with the ability to enhance nutrition efficiency, abiotic stress tolerance, and plant quality traits [20]. The European Biostimulants Industry Council (EBIC) supports the usage of plant stimulants as a reliable solution to secure the high yield and good quality of crops, while, at the same time, using the available resources wisely [21]. The European Parliament also launched Regulation (EU) 2019/1009, with the aim to achieve fertilizer usage regulation and harmonization of the market for the production of these products, and it is of special interest since, for the first time, microbial-based biostimulants were recognized within a group of organic fertilizers [22]. Microbial plant biostimulants are considered as an important group of plant stimulants with beneficial microorganisms as active components which, through several mechanisms of action, are capable of enhancing plant nutrition efficiency, stress tolerance, and the induction of systemic resistance against plant pathogens, while also contributing to the suppression of plant diseases. Previous research in this field has indicated that representatives of the *Azotobacter*, *Rhizobium*, or *Sinorhizobium* genus fix atmospheric nitrogen, while the *Trichoderma* or *Pseudomonas* genus members are of particular interest for phosphorous solubilization, and the *Acidothiobacillus*, *Paenibacillus*, and *Bacillus* species have the ability to perform potassium solubilization [22]. Among the several genera with potential biostimulant action, representatives of the *Bacillus* genus are of the most interest for the agro-biotech industry. The commercialization of *Bacillus*-based products intended for usage in agriculture has a growing trend worldwide. Apart from the beneficial activities regarding biostimulant and biocontrol effects, *Bacillus* strains are able to persist under various environmental conditions and remain stable and viable during distribution, storage and application in the field. The ability to grow rapidly in different cultivation media due to low nutritional requirements and the production of various metabolites and endospores contribute to their suitability to be used as microbial active components in plant biostimulant products [20].

Taking into account all the aforementioned research, the aim of this study was to investigate the composition of strawberry juice production wastewater (SJPW), in terms of antioxidant content, to assess the feasibility of this waste stream to recover antioxidants as value-added chemicals. Furthermore, an additional valorization route, based on the microbial conversion to plant biostimulant, was investigated, to propose a viable lab-scale solution for the usage of SJPW in the concept of a circular bioeconomy. Strawberries are grown on almost 7000 ha of arable land in the Republic of Serbia, with an estimated yield of 3.2 t/ha, providing an overall output of around 21,800 t/year [23]. Almost two-thirds of these strawberries are being exported, while the rest is used for consumption as both fresh or frozen strawberries or processed into various products, of which strawberry juice is the most common, generating a significant load of processing wastewater that has been investigated in this study as a circular resource.

## 2. Materials and Methods

### 2.1. Origin and Collection of Strawberry Juice Production Wastewater (SJPW)

The SJPW was the waste material collected from the washing of production tanks during strawberry juice production in 2022. Wastewater from the production of strawberry juice was taken from the production plant in Serbia, Nectar d.o.o., which deals with the production of products made from fruits, such as juices, nectars, alcoholic beverages and jams. The strawberries were of the Clery variety, grown in the vicinity of the village Bač, Serbia, in 2022. The technological process of strawberry juice production includes reception, washing, and inspection of fruits, rough mashing, blanching, fine mashing, storage and pulp correction, pasteurization, filling in aseptic conditions, and storage. During the pasteurization of the strawberry mash, water is added to the tank with the mash so that the mash can be passed through the pasteurizer. After pasteurization, the residual mixture of water and mash in the tanks and pipelines is separated from the system as wastewater—strawberry juice production effluent. This effluent was collected as samples for this study and immediately frozen at −20 °C until usage.

### 2.2. Analysis of the Basic Nutritional Profile of SJPW

The basic nutritional profile of the SJPW was determined by applying several methods and techniques to determine dry weight, total sugar and reducing sugar content, protein content, and cellulose (crude fiber) content. Dry weight was measured refractometrically using Abbe’s universal refractometer by directly reading the measurement results from the refractometer scale (Official Gazette of SFRY, No. 29/83) [24]. The content of total sugars was analyzed according to the Luff–Schoorl method (Official Gazette of SFRY, No. 29/83). All measurements were repeated three times, and the results were expressed as the mean value in percentages. Determination of reducing sugar content was performed by applying the volumetric method (Official Gazette of SFRY, No. 29/83). The protein content was determined by the Kjeldahl method [25]. The content of cellulose (crude fiber) was determined by the Kirschner–Ganakova method [26].

### 2.3. Analysis of Antioxidants’ Content of the Extracts of SJPW

To prepare the SJPW extract, 100 mL of wastewater was mixed with 100 mL of methanol. The mixture was then subjected to maceration for 24 h at room temperature on a shaker set to 160 rpm. After maceration, the extracts were filtered through filter paper and the solvent was evaporated under reduced pressure at 40 °C. Once the extracts were dry, they were dissolved in 50% methanol to achieve a final concentration of 100 mg/mL. Extract was prepared in triplicate. This procedure was adapted from Simin et al. [27], with some modifications to account for the different nature of the raw material.

#### 2.3.1. LC–MS/MS Analysis of Selected Phenolic Compounds in the SJPW

The levels of quinic acid and 44 selected phenolic compounds were analyzed using LC–MS/MS, following the method described by [28]. The analysis was conducted with a Series high-performance liquid chromatograph (Agilent Technologies 1200, Santa Clara, CA, USA) connected to a Triple Quad tandem mass spectrometer equipped with an electrospray ion source (Agilent Technologies 6410A, Santa Clara, CA, USA). The system was operated using the MassHunter Workstation software—Data Acquisition (version B.06.00) (Agilent Technologies). Extracts were initially diluted to a concentration of 20 mg/mL with 50% aqueous methanol and further diluted as needed. A 5 μL sample was injected into the system, and compounds were separated using a Zorbax Eclipse XDB-C18 rapid resolution column (50 mm × 4.6 mm, 1.8 μm). Data were collected in the dynamic MRM mode, and peak areas were analyzed with the Agilent MassHunter Workstation Software—Qualitative Analysis (version B.06.00). Calibration curves, generated using the OriginLabs Origin Pro (version 2019b) software, were used to calculate the concentrations of the compounds in the extracts. All analyses were performed in triplicate, and results were reported as micrograms of each compound per gram of dry extract.

#### 2.3.2. Determination of Total Phenolic Content in the SJPW

Total phenolic content was measured using the Folin–Ciocalteu (FC) reagent, following the method outlined by Lesjak et al. [29]. The methanol extract was tested at concentrations of 1.25, 2.5, and 5.0 mg/mL, with all assays conducted in triplicate. Results are reported as milligrams of gallic acid equivalents (GAE) per gram of dry extract (mg GAE/g de) and milligrams of gallic acid equivalents per liter of SJPW (mg GAE/L SJPW).

#### 2.3.3. Determination of Total Flavonoid Content in the SJPW

Total flavonoid content was assessed using the colorimetric method detailed by Lesjak et al. [29]. The methanol extract was evaluated at concentrations of 1.25, 2.5, and 5.0 mg/mL, with all the measurements conducted in triplicate. The results are reported as milligrams of quercetin equivalents per gram of dry extract (mg QE/g de) and milligrams of quercetin equivalents per liter of SJPW (mg QE/L SJPW).

### 2.4. Estimation of Antioxidant Potential of Extracts from the SJPW

#### 2.4.1. DPPH Assay

The capacity of the extracts to neutralize DPPH radicals was evaluated using a modified method based on Mimica-Dukic et al. [30] and tailored for microplate assays. The extracts were tested in concentrations ranging from 0.625 to 40.0 mg/mL, combined with 100 µL of a 67.5 µM DPPH solution in methanol and 190 µL of methanol. The results are reported as the IC50 value, which indicates the concentration needed to neutralize 50% of the DPPH radicals (mg/mL). IC50 values were calculated by generating inhibition–concentration curves with OriginLabs Origin Pro (version 2019b) software. The percentage of inhibition achieved by the different extract concentrations was calculated with the following equation:

I(%) = (1 − A/A0) × 100(1)

where A is the absorbance of the investigated extracts corrected by the absorbance of the blank probe, and A0 is the absorbance of the control.

Results are expressed as IC_50_ value (the concentration of the sample that neutralizes 50% of DPPH^•^ (mg/mL)). The IC_50_ value was determined by plotting inhibition–concentration curves in the OriginLabs Origin Pro (ver. 2019b) software.

#### 2.4.2. FRAP Assay

The Ferric Reducing Antioxidant Power (FRAP) assay was conducted following the procedure outlined by Lesjak et al. [29]. The methanol extract of SJPW was evaluated at concentrations of 1.25, 2.5, and 5.0 mg/mL. All assays were performed in triplicate, and the results are presented as milligrams of ascorbic acid equivalents per gram of dry extract (mg AAE/g de).

### 2.5. Microbial Active Component

The strain *Bacillus* sp. BioSol021 was applied as a microbial active component in this study. The strain was isolated from the rhizosphere of a bean (*Phaseolus vulgaris*) [31] and previously identified as the member of the operational group *Bacillus amyloliquefaciens* by 16S rRNA gene sequencing. The 16S rRNA gene sequence is deposited in the NCBI (National Center for Biotechnology Information) GenBank under the accession number ON569805 [32].

### 2.6. Biostimulant Production—Cultivation Conditions and Monitoring

The inoculum of the microbial active component was prepared using the synthetic liquid medium nutrient broth (Himedia Laboratories, Thane, India), while the SJPW was sterilized by autoclaving and used as a medium in the cultivation phase without any further treatment aside from sterilization. Cultivation of the microbial active component was carried out in the 16 L-lab-scale bioreactor (EDF–15.4_1, A/S Biotehniskais center, Riga, Latvia), with simultaneous in-line sensor-based monitoring of temperature, pH value, and DO (dissolved oxygen) content and offline monitoring of biomass and sugar content. Temperature was regulated at 28 °C using the bioreactor-integrated temperature control system, while the pH value was initially measured at 4.85, set to 7.0 using 1 M NaOH; it was not further adjusted during the cultivation, only monitored using the pH sensor. As for the aeration rate, it was maintained at 1 vvm (volume of air per volume of liquid per min) throughout the cultivation, which lasted 96 h. Agitation rate was set to 100 rpm using the Rushton three impeller turbine. Biomass content of *Bacillus* sp. BioSol021 was determined using the plate count method, while the HPLC–RID (refractive index detector) method [33] was employed to determine sugar content at predefined sampling intervals. Experimental sets performed in the bioreactor were conducted in three repeats, and mean values were calculated and further analyzed. Visualization of cultivation course monitoring was performed using the LabPlot v. 2.9 software (github.com/KDE/labplot).

### 2.7. In Vitro PGP (Plant Growth Promotion) Traits Screening of the Produced Biostimulant

Cultivation broth samples obtained after 96 h of cultivation were used to investigate the in vitro PGP (plant growth promotion) traits of the microbial active component *Bacillus* sp. BioSol021. The following PGP traits were investigated: production of IAA (indole acetic acid—Vlajkov et al., 2022) [34], production of ACC (1-aminocyclopropane-1-carboxylate) deaminase (Penrose and Glick, 2003) [35], as well as hydrolytic enzymes facilitating plant nutrition—protease, xylanase, cellulase, and pectinase [34] (Vlajkov et al., 2022). The raw juice wastewater was used as a negative control. Analysis and visualization of the experimental data related to enzymatic activity were performed using the Statistica 13.2 software (Dell, TX, USA). Duncan’s multiple range test was applied to define homogenous groups of variances of the dependent variables. All statistical analyses were performed at the significance level of 95%.

### 2.8. PGP Activity of the Biostimulant in the Seed Germination Phase

The ability of the value-added biostimulant to promote the growth of tomatoes (*Solanum lycopersicum*) in the initial growth phases was investigated by applying the seed germination test. Briefly, 20 tomato seeds were placed on filter paper at the bottom of a Petri dish and treated with 1 mL of *Bacillus* sp. BioSol021 cultivation broth and 3 mL of tap water, while 4 mL of tap water was used in control samples. The raw juice wastewater was also used for negative controls, prepared in the same way as samples. The incubation of the seeds was carried out in the dark for 7 days at room temperature. After that, the number of germinated seeds was determined by counting, simultaneously with root and stem length measurement. Data analysis and visualization in this experimental phase were performed using the Statistica 13.2 software (Dell, TX, USA). Duncan’s multiple range test was applied to define homogenous groups of variances of the dependent variables. All statistical analyses were performed at the significance level of 95%.

## 3. Results

The initial investigation step included the determination of the SJPW basic nutritional profile presented in Section 3.1. This was followed by its antioxidants’ content and antioxidant potential assessment shown in the Section 3.2. The third part of the present research included the monitoring of the plant biostimulant production performed in a lab-scale bioreactor (Section 3.3), and the final step presented the evaluation of the PGP ability of the microbial active component (Section 3.4).

### 3.1. Basic Nutritional Profile of SJPW

The basic nutritional profile of SJPW was investigated in terms of dry weight, total sugar and reducing sugar content, protein content and cellulose (crude fiber) content, as well as pH value, to assess its suitability as a raw material in microbial-based bioconversion processes. The results obtained regarding the basic nutritional profile of SJPW are given in Table 1.

### 3.2. Antioxidant Content and Antioxidant Potential of SJPW

LC–MS/MS method was employed to determine the content of 44 selected phenolic compounds (phenolic acids, flavonoids, coumarins, and lignans) as well as quinic acid in SJPW. Out of the 45 analyzed compounds, 14 were quantified, 6 were present at concentrations below the instrument’s limit of quantification, and 26 were not detected. Analyzed but not detected were the following compounds: cinnamic, gentisic, *o*–coumaric, ferulic, syringic, 3,4–dimethoxycinnamic and sinapic acid, daidzein, genistein, apigenin, apigenin–7–*O*–glucoside, baicalin, baicalein, epicatechin, epigallocatechin gallate, kaemferol, luteolin, myricetin, vitexin, amentoflavon, apiin, esculetin, umbeliferon, matairesinol and secoisolariciresinol. The obtained results are presented in Table 2.

The antioxidant potential of SJPW was investigated using the DPPH (2,2-diphenyl-1-picrylhydrazyl) and FRAP (ferric reducing antioxidant potential) assays, while the total phenolic and flavonoid contents of SJPW were investigated using spectrophotometric methods. The results of the aforementioned assays are given in Table 3.

### 3.3. Course of Bacillus sp. BioSol021 Cultivation Using SJPW as a Medium

**Figure 1:**
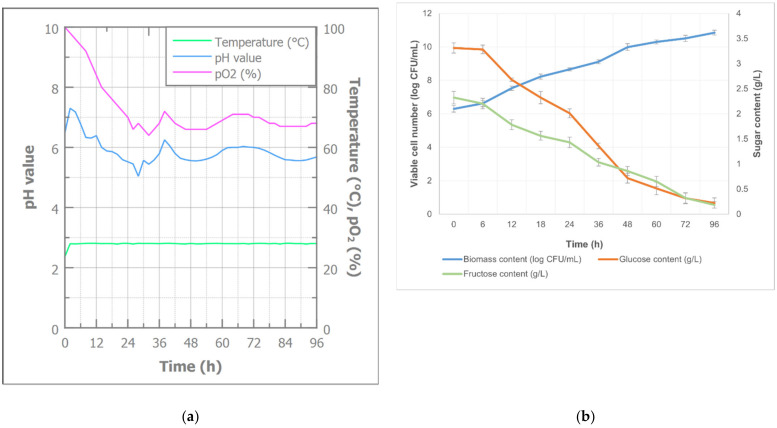
Cultivation course of biostimulant production using SJPW as a medium: (**a**) bioprocess parameters during *Bacillus* sp. BioSol021 cultivation; (**b**) biomass and sugar content during *Bacillus* sp. BioSol021 cultivation.

Cultivation of *Bacillus* sp. BioSol021 using SJPW as a medium aimed at plant biostimulant production was carried out in a 16 L-lab-scale bioreactor for 96 h. The cultivation course was monitored in-line in terms of bioprocess parameters (temperature, pH, and pO_2_—as given in Figure 1a), as well as off-line to determine the biomass content (viable cell number) and sugar content at predefined sampling intervals, as given in Figure 1b.

### 3.4. PGP Traits of Microbial Active Component

**Figure 2:**
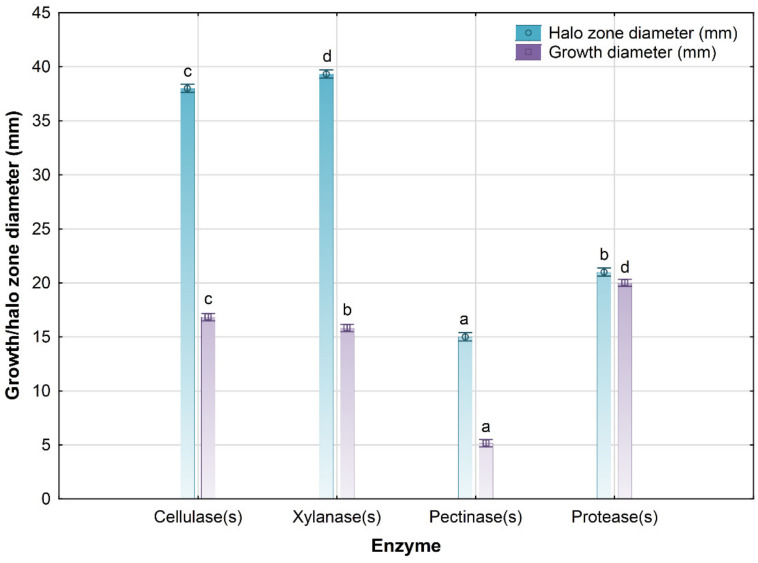
Enzymatic activity in terms of PGP traits of microbial active component *Bacillus* sp. BioSol021. Letters (a–d) represent different levels of statistical significance. Values marked with the same letter are at the same level of significance.

The microbial biomass contained in the cultivation broth samples obtained after the cultivation of *Bacillus* sp. BioSol021 using the SJPW as the medium in the lab-scale bioreactor was further examined for its in vitro PGP traits in terms of IAA production and enzyme activity. The achieved level of IAA production is 8.55 ± 0.05 mg/L, while the results regarding enzymatic activity are presented in Figure 2. The following mean values of enzymatic activity index (EAI) are achieved: 2.26 ± 0.04 for cellulolytic activity, 2.49 ± 0.08 for hemicellulolytic activity, 2.91 ± 0.16 for pectinolytic activity, and 1.05 ± 0.00 for proteolytic activity.

### 3.5. PGP of the Microbial Biostimulant in the Initial Growth Phases of Tomato

**Figure 3:**
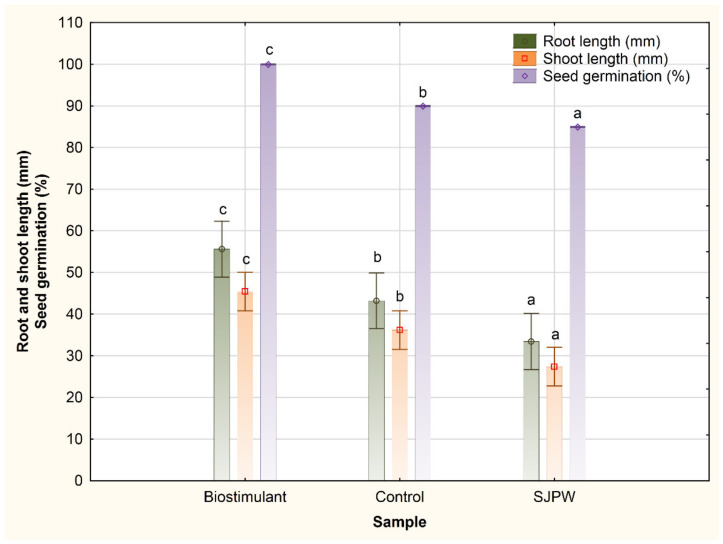
Tomato root and shoot length promotion using the biostimulant based on *Bacillus* sp. BioSol021, produced using SJPW as the cultivation medium in the lab-scale bioreactor. Letters (a–c) represent different levels of statistical significance. Values marked with the same letter are at the same level of significance.

The biostimulant produced using SJPW in the lab-scale bioreactor was examined for growth promotion of tomatoes in the initial growth phases following the seed germination. Indeed, the germination percentage of tomato seeds was maximal (100%) when the biostimulant was applied, while the germination percentage of control seeds was lower (90%). Furthermore, the root and shoot lengths of the newly formed seedlings were recorded after 7 days of incubation, and the results are given in Figure 3.

## 4. Discussion

Depending on the fruit variety, the ratio of strawberry juice yield ranged from 48.22% to 89.98% compared to fresh fruit mass [36]. During fruit processing, a majority of the insoluble components remains in the pomace, while the soluble sugar compounds dominantly end up in the juice. Therefore, a significant amount of sugars in the wastewater from the strawberry juice storage tanks was expected to be obtained before pasteurization. Regarding the nutritional requirements of the bacteria from the *Bacillus* genus, which rely on a carbon source in the form of sugars, phosphorus, and nitrogen, the results obtained in terms of SJPW nutritional content indicate that no adjustments were required, except for the pH value, which was initially set to 7.0 ± 0.2 before the medium sterilization by autoclaving. Generally, the pH value of the fruit juice production wastewater is usually observed to be in the range of 3.85–8.00 [2,37,38].

When it comes to antioxidant properties, fresh strawberries exhibit an antioxidant potential 2 to 10 folds higher compared to other fruits, such as apples, peaches, oranges, and kiwifruits [39]. The Clery variety is characterized by a total phenolic content of 55.1 ± 1.7 mg/100 g FW to 79.3 ± 4.0 mg/100 g FW, depending on the fruit size [37]. Similar results were observed when growing the same strawberry variety in the climate conditions in Serbia, where the application of microbial products based on *Bacillus* strains has significantly improved the total phenolic content in strawberries by 20% [40]. However, a majority of the phenolic compounds from fresh fruit is lost during juice production, considering that a large amount of the phenolic compounds remains bound to the cell wall material [41]. This was also confirmed in the study by Oszmiański and Wojdyło (2009) [42], where the highest loss of phenolic compounds and, consequently, the lowest antioxidant potential were observed in clear strawberry juice compared to cloudy and pureed juices. We thus expected to observe an even lower content of phenolic compounds in SJPW, considering the dilution of the strawberry juice during the processing, as well as the removal of suspended solids and the bound phenolics with the juice fraction. Total phenolic content in the strawberry juice obtained by blanching and homogenization was 12.84 ± 0.03 mg GAE/g de and 11.97 ± 0.10 mg GAE/g de for the samples undergoing mixer homogenization and homogenization by the T18 Ultraturrax^®^ homogenizer, respectively, considering the initial sample mass of 5 g that was used for the extraction of the phenolics [41]. A slightly lower phenolic content was observed in this study (8.52 ± 0.37 mg GAE/g de), considering the initial sample volume of 100 mL used for the extraction of the phenolics. The observed phenolic content of 54.4 mg GAE/L was higher compared to the grape juice industry wastewater (5.4 mg GAE/L—Poblete et al., 2021) [42] and lower compared to the pear and apple processing wastewater (512 mg GAE/L—Amor et al., 2012) [3]. On the other hand, the observed content of flavonoids in SJPW was 0.48 ± 0.03 mg QE/g de. Garzoli et al. (2020) [43] had observed the total flavonoid content of 0.96 ± 0.05 mg rutin equivalents/g (RE/g) de and 1.08 ± 0.05 mg RE/g de for the strawberry juice samples which underwent blanching and homogenization by mixer and the T18 Ultraturrax^®^ homogenizer, respectively. The aforementioned results emphasize approximately 35–40% lower phenolic and flavonoid contents in the liquid effluents of strawberry juice production compared to the juice products themselves. When considering the total phenolic and flavonoid contents in comparison to the volume of strawberry juice, a phenolic content of around 560 mg GAE/L and flavonoid content of around 440 mg RE/L were observed by Chen et al. (2023) [44], which are higher than those observed in the SJPW in this study.

When lowering the observation scale to the individual antioxidant compounds, similar results could be observed. For example, the observed content of *p*-coumaric acid (in mg/L) in clear, cloudy, and pureed strawberry juice was in the range of 24.2 ± 0.2 to 29.4 ± 0.2, 25.7 ± 0.9 to 34.9 ± 0.4, and 28.9 ± 1.9 to 35.9 ± 1.1, respectively [42], which was significantly higher than the *p*-coumaric acid content observed in the SJPW in this study (16.2 ± 1.46 µg/L). Similar results were also obtained for other the representative antioxidant compounds, e.g., catechin, which was present in the SJPW in the amount of 180 ± 18.0 µg/L, while significantly higher amounts were observed in the clear (14.0 ± 2.3 to 20.3 ± 0.4 mg/L), cloudy (13.4 ± 1.2 to 23.4 ± 1.6 mg/L), and pureed strawberry juices (15.8 ±0.8 to 25.6 ± 2.4 mg/L) [41]. On the other hand, quercetin and kaempferol content was below the instrument’s quantification and detection limits, respectively, when it came to the SJPW, while their content in different strawberry juice fractions was in the range of 1.5–12.3 mg/L [42]. The higher affinity of the antioxidant compounds for suspended solids in the strawberry juice, followed by a dilution factor, i.e., the addition of fresh water during strawberry processing, resulted in a very low content of individual antioxidant compounds in the SJPW, which was also reflected in their lower antioxidant potential. The ability of the SJPW extracts to neutralize DPPH• was measured as the IC50 value of 0.69 ± 0.03 mg/mL. Patras et al. (2009) [45] determined the DPPH scavenging activity for strawberry juice to be in the range of 1.16–1.65 mg/mL, expressed as ARP (anti-radical power; the inverse of the IC50 value) which was comparable with the SJPW extract (ARP—1.44 mg/mL) [44]. On the other hand, Chaves et al. determined the DPPH scavenging activity of strawberry extracts as IC50 to be in the range of 76.7–110 mg/mL, which are multiple times higher than the IC50 for the SJPW extract [46]. Additionally, Simirgiotis et al. (2010) reported an IC50 for strawberry fruit extract of 38.7 ± 0.80 µg/mL, which is about twenty times lower than the value for SJPW [47]. On the other hand, the FRAP of the SJPW extracts was 4.43 ± 0.28 mg of AAE/g de, while it was estimated at 14.4 mol of Fe^2+^ equivalents/L for strawberry juice [48]. The FRAP was also previously estimated for different types of strawberry juices, i.e., clear juice (335.39–470.82 µM of Trolox/100 mL), cloudy juice (380.11–514.67 µM of Trolox/100 mL), and pureed juice (957.38–1155.86 µM of Trolox/100 mL) [41]. Cao et al. reported the antioxidant activity of strawberry juice to be in the range of 450–750 mg AAE/L juice, multiple times higher than the value for SJPW which is 28.4 ± 1.79 mg AAE/L [49]. The different modes of expressing antioxidant properties (activity, capacity, and potential), as well as the different methods applied for the determination of antioxidant properties, call for a standardization of methods in terms of the experimental conditions applied and the expression of results to provide better comparability of experimental results among the published studies in this field, as well as for more precise nomenclature of the antioxidant properties [50,51].

The presented results in terms of antioxidant compound content point out the low potential of SJPW as a raw material for the possible recovery of compounds with antioxidant potential. Hence, another valorization route aimed at its utilization as a raw material in microbial bioconversion to plant biostimulants was investigated. A better understanding of the observed bioprocess including the cultivation of *Bacillus* sp. BioSol021 in the lab-scale bioreactor is enabled by the observation of key bioprocess parameters, including temperature, pO_2_ and pH levels, as well as biomass content and concentrations of glucose and fructose as the main nutrients (carbon sources) contained in the SJPW.

Figure 1a presents the trend of the pO_2_, temperature, and pH value changes during the 96 h long cultivation, pointing out a continuous decrease in the amount of available oxygen. It can be observed that the oxygen consumption is in line with the biomass growth (Figure 1b), and it is obvious that the drop in the oxygen content is associated with the moment when the microbial cells enter the intensive growth phase characterized by the higher oxygen demand [33]. Further fluctuations in the oxygen level during the stationary growth phase are of evidently lower intensity and a consequence of the changes in the metabolic activity rhythm. The same figure (Figure 1a) shows the pH level changes, indicating an intensive downward trend in the first 24 h of bioprocess, with a similar profile compared to the previously described oxygen level concentration following the exponential growth of bacterial cells. Since the next phase is characterized by intensive metabolic activity, it can be observed that the production of acidic and basic compounds was dominant in different stages of the stationary phase, resulting in pH level fluctuations. However, the overall changes in the pH level during the entire bioprocess were close to the optimal neutral range for the producing microogranism, without the necessity for its regulation, which was also confirmed by the biomass growth trendline (Figure 1b). The lowest pH value was reached after 28 h of cultivation (5.04), while the highest value was observed in the fourth hour of cultivation (7.18). The initial pH value of the cultivation medium was slightly lower (6.48) than initially adjusted before sterilization (7.0), which was expected due to the nutrients’ chemical interactions at increased temperature and pressure. The resulting cultivation broth after the end of the bioprocess had a pH value of 5.68, which was also suitable for plant applications considering the pH values of other commercially available biostimulants [52,53].

The biomass content changes were also monitored during the bioprocess, and the obtained trend is shown in Figure 1b. The initial growth phase was characterized by the lag phase that lasted less than 12 h, indicating good adaptation of the producing microorganism *Bacillus* sp. BioSol021 to the cultivation conditions and adequacy of the applied medium [2]. The growth curve indicates a drastic increase in microbial cells concentration in the first 24 h, as a typical phase of exponential growth, followed by further increase in biomass content but with a lower intensity. The rapid replication of cells slowed down after 48 h, when the metabolic activity in secondary metabolism became dominant. The rising growth trend of biomass concentration continued until the end of the bioprocess, reaching the maximal level of 10.84 log CFU/mL, which was almost double the value compared to the beginning of the cultivation.

The monitoring of the nutrient level is observed by the measurement of glucose and fructose content and presented in Figure 1b. The dynamics of sugar content changes can be observed through the phases of the previously described bacterial growth curve. The first hours of cultivation were characterized by a lower intensity of nutrient consumption as a consequence of the adaptation of the microbial cells to the applied cultivation conditions. An obvious decrease in the content of the both carbon sources started when the exponential growth phase had been initiated. However, more rapid consumption in the first 48 h was observed in the case of glucose, implying higher affinity of the producing microorganisms towards this carbon source. Both nutrients were almost completely utilized after 96 h, with consumption levels of 93.24% and 92.08% for glucose and fructose, respectively. The results obtained point out the potential of the proposed bioprocess based on *Bacillus* sp. BioSol021 to be used as an efficient valorization route for the SJPW.

The use of fruit processing industry effluents and by-products in the biorefinery concept has, so far, been mostly investigated in terms of nutrient recovery [1,54] and less in terms of microbial bioconversion into value-added biotechnological products. For example, single-cell protein production was investigated simultaneously with the biological treatment of fruit juice industry wastewater [2]. Pulp wash obtained from the residues of orange juice extraction was investigated as a raw material for the production of *Bacillus thuringiensis* bioinsecticide [55]. Solid fruit processing effluents were also investigated as nutrient sources for *Bacillus* strains cultivation, aimed at exopolysaccharide production [56], lipopeptides (surfactin and fengycin) production [57], laccase production [58], and biohydrogen production [59]. To the best of the authors’ knowledge, this is the first study exploring the possibility of SJPW valorization as the cultivation medium for the production of *Bacillus*-based biostimulants.

Biostimulant activity was first tested in vitro to assess the PGP traits of the produced cultivation broth based on *Bacillus* sp. BioSol021 and SJPW. The observed level of IAA production was 8.55 ± 0.05 mg/L, which was slightly higher than what was produced by several *Bacillus* strains in the study by Cabra Cendales et al. (2017) [60], which was in the range of 2.49–5.47 µg/mL. Both lower (1.9 µg/mL) and higher levels (49.39 µg/mL) of IAA production by several *Bacillus* strains were reported in the study by Kouam et al. (2023) [61], as well as in the study by Patani et al. (2023) [62] (5.3 µg/mL for *Bacillus safensis*; up to 45.7 µg/mL for *Bacillus firmus*). IAA is primarily responsible for root elongation by increasing cell division and the differentiation of tissue, further resulting in increased plant fresh mass [60], thus its production is one of the main PGP traits to be investigated for the potential active components of biostimulants. IAA production was also previously investigated in relation to *Bacillus* sp. BioSol021, the biostimulant active component applied in this study, using the commercial synthetic cultivation medium in our previous study [34], and it was found to be slightly lower compared to the SJPW medium (6.76 ± 0.04 mg/L).

Enzymatic activity of the PGP strains is an important trait, contributing to enhanced and facilitated plant nutrition, improved nutrient availability in the rhizosphere for other microflora constituents, and improved soil biodiversity [63,64]. In this study, the in vitro activities of the cellulase(s), xylanase(s), pectinase(s), and protease(s) produced by the *Bacillus* sp. BioSol021 were investigated to assess the potential of the biostimulant to contribute to more efficient nutrient utilization, considering the major nutrients arising from the plant residues available in the soil and thus also contributing to the improvement in soil quality. Mean values and standard deviations of the achieved enzymatic activities are presented in Figure 2 and grouped using Duncan’s multiple range test in homogenous groups of the same statistical significance. The highest level of enzymatic activity was observed in the cases of the xylanase(s) and cellulase(s), with halo zone diameters over 35 mm, indicating substrate (SJPW)-induced enzyme production by the *Bacillus* sp. BioSol021 during the biostimulant cultivation process, as well as a high level of biomass enzymatic productivity, given that biomass growth diameters over 15 mm were observed in the case of both xylanase(s) and cellulase(s), indicating high values of EAI (over two in both cases). Extracellular pectinase(s) activity showed dominance, with the highest value of EAI (2.91 ± 0.16) among the investigated enzymes. A similar situation regarding the pectinolytic activity was observed during the investigation of enzymatic activity of *Bacillus* sp. BioSol021 cultivated using nutrient broth as the synthetic medium [34], while a significant improvement in xylanolytic activity was achieved when using the SJPW medium, suggesting the significance of the presence of hemicellulose fibers as substrates inducing xylanase(s) production in SJPW. The negative control, SJPW, showed no enzymatic activity.

Considering the biostimulant effect on tomato growth, many *Bacillus* strains were investigated both for the improvements in tomato germination as well as seedling growth in later growth stages, such as *Bacillus subtilis* GIBI 200 [60], *Bacillus subtilis* MBI600 [64], *Bacillus thuringiensis* B9 and *Bacillus pacificus* B11 (Kouam et al., 2023), *Bacillus velezensis* 83 [65], etc. *Bacillus* sp. BioSol021 was previously investigated and confirmed as a biostimulant in the case of corn seed germination, both as a liquid biostimulant produced using a semi-synthetic medium [34] and a soil amendment as a biochar-immobilized active component [66]. Regarding the tomato growth promotion observed in this study, the complete germination of the tomato seeds when applying the produced biostimulant had a 10% increase in comparison to the control, with only water applied to provide sufficient humidity for seed germination. It is a significantly higher germination percentage compared to the studies using *Bacillus subtilis* GIBI 200 (86.7%—Cabra Cendales et al., 2017) [60], *Bacillus* sp. NCT4 (95%—Patani et al., 2023) [62] or *Bacillus thuringiensis* B9 (88%—Kouam et al., 2023) [61]. Furthermore, a significant increase in tomato root and shoot growth promotion was achieved in this study—29% and 26%, respectively—with mean root length of 55.60 ± 11.32 mm and mean shoot length of 45.45 ± 3.71 mm after 7 days, significantly higher compared to the control (43.20 ± 18.08 mm and 36.20 ± 12.76 mm) based on Duncan’s multiple range test. A significantly higher value of tomato shoot length was observed after 7 days using *Bacillus* sp. BioSol021 as the biostimulant active component compared to the combination of the *Bacillus* and *Pseudomonas* strains (26.85 mm—Widnyana and Javandira, 2016) [67]. Comparable results in terms of tomato growth promotion in the initial growth phases were obtained in the case of *Bacillus subtilis* subsp. *spizizenii* [68]. Previous research has indicated that *Bacillus* isolates with the PGP ability promote plant growth by different mechanisms, including hormone production such as auxin and IAA which are supplied to the host, or by regulating the expression of auxin-related host genes, resulting in increased auxin production [69]. The isolate of interest used in the present study, *Bacillus* sp. BioSol021, was not only previously reported as a significant producer of IAA but also as a surfactin-producing strain, exhibiting biocontrol effect as an indirect PGP mechanism. The promising potential of *Bacillus* sp. BioSol021 was previously observed regarding the production of key hydrolytic enzymes, including cellulases, xylanases, pectinases, and proteases, which is important from the aspect of plant nutrition facilitation, overall crop cultivation improvement by rhizosphere nutritional enrichment, and soil quality improvement. *Bacillus* sp. BioSol021 has shown a positive result in terms of the ability to solubilize phosphates, produce ammonia and ACC deaminase, and contribute to plant stress alleviation, resulting in improved plant growth [33,66]. Further investigation steps will include a detailed analysis of the broader metabolic profile involved in plant–microbe interaction, including an investigation of the multiple plant host-specific responses, as well as genomic-based research to indicate the presence of the key genes involved in plant growth promotion action. Additionally, it is important to mention that the SJPW application in the germination tests indicated the inhibition effect on plant growth compared to the control group and the *Bacillus*-based plant biostimulant, which also has to be studied further to identify the potential plant growth inhibitors. However, it is clear that application of the SJPW without proper pretreatment should not be considered for agricultural applications considering the following two different aspects: (a) ecological, due to the high organic matter content of the effluent that needs to be addressed/removed prior to disposal in the environment and (b) the negative impact on the tomato root and shoot length, with significantly lower values compared to the tap water used as a control sample (25% lower value for shoot and 23% for root length), indicating a necessity to further investigate the potential inhibitory compounds in the SJPW, as well as a wider range of plant host responses. Considering the SJPW composition represented in Table 1, its application for agricultural purposes without proper pretreatment could also be concerning from the aspect of potential eutrophication effects [70] and confirms the necessity for appropriate effluent processing before agricultural/natural water recipient discharge, minimizing the risk of consequent environmental problems. With this in mind, the fact that the cultivation broth (biostimulant) samples did not show inhibition activity on tomato seed germination and plant growth in the initial growth phases but did exhibit PGP effects can be observed as an additional benefit of the designed bioprocess solution for biostimulant production, due to the suppression of the negative effect of the raw juice wastewater on plant development. Thus, the proposed “biological treatment” of the SJPW resulted not only in the value-added biostimulant product but also confirmed the potential of the proposed microbially mediated wastewater treatment and valorization route in increasing the applicability of this specific industrial effluent in subsequent agricultural applications.

## 5. Conclusions

Considering all the results presented and discussed in this study, it can be concluded that SJPW could be further investigated as a valuable circular resource in the food industry and the inter-related agricultural production. Although, based on the results of the present study, the usage of SJPW for antioxidant recovery is questionable, it could still be applied as a suitable substrate for the production of plant biostimulants. The results of studies based on other feedstock usage have indicated higher concentrations of the obtained compounds; however, a detailed techno-economic analysis has to be performed to evaluate the potential for large-scale antioxidant recovery using the proposed waste material. On the other hand, considering that significant growth promotion of tomato was achieved in this study in the initial growth phases, without any signs of plant growth inhibition, the following research activities should also focus on other crops, as well as on greenhouse and field experiments, to estimate biostimulant activity in real application conditions.

Wider commercialization of the plant biostimulant production is directly influenced by the scalability potential of the proposed bioprocess solution. Technology transfer to an industrial scale is a challenging step that requires a detailed techno-economic analysis to define the optimal parameters for production, aimed at the maximization of biomass and bioactive metabolite content as well as enzymatic activity. The stage of bioprocess development included in the present study is a necessary precondition for designing an industrially viable solution, considering that the cultivation was performed at the level of a laboratory bioreactor. Future research steps will include experimental sets addressing bioprocess parameter optimization, followed by a further scale-up of the defined solution to the pilot and industrial levels. The focus of the upcoming investigation will be defining the optimal values of pH value, temperature, and agitation and aeration rates, based on the generated mathematical models integrated with the commonly used optimization methods. A further scale-up of the bioprocess solution developed at the laboratory level, classified as Technology Readiness Level 4 (TRL 4), should provide a better understanding of the bioprocess specificities through a detailed characterization of the kinetics (including microbial growth, nutrient consumption, specified metabolites production) and engineering/design parameters, including the scale and geometry of the bioreactor vessel and the mixing and aeration elements, affecting mass and energy transfer and hydrodynamics as an important precondition for maximizing target bioprocess outputs at the higher production stage. Existing techno-economic questions regarding the feasibility of the proposed bioprocess solution, considering the high cost of the initial investment characteristics for biotechnological production, could be addressed through software simulations for industrial applications, providing profitability insights and bringing the transfer of the designed technology closer to the commercialization stage. One of the hypotheses would be that the application of an industrial waste-based medium would contribute to the overall bioprocess techno-economical effectiveness and lower the production cost of the plant biostimulant compared to synthetic media commonly applied in the production of biostimulants and the related biological products for agricultural applications; however, this hypothesis must be verified in subsequent bioprocess modeling and simulation steps to be included in the following research activities.

Additionally, other food industry effluents could also be investigated for value-added biostimulant production purposes; this will also be one of the research directions to be pursued in the future, opening a new chapter of possibilities for circular bioeconomy advancement by unlocking the hidden potential of waste while reducing the environmental burden and footprint arising from food industry effluents.

## Figures and Tables

**Table 1:** The basic nutritional profile of strawberry juice production wastewater (SJPW).

Parameter	Value (Unit)
Dry weight	0.65 °Bx
Sugar content	5.60 g/L
Reducing sugar content	2.70 g/L
Protein content	2.02 g/L
Cellulose content	1.13 g/L
pH value	4.85

**Table 2:** Content of quantified phenolic compounds in SJPW.

Compound	Content (µg/g de) ^a^	Content (µg/L SJPW)
Organic acids		
Quinic acid	3102 ± 310	19,852 ± 1985
Phenolic acids		
p–Hydroxybenzoic acid	6.62 ± 0.40	42.4 ± 2.54
Protocatechuic acid	25.9 ± 2.07	166 ± 13.2
p–Coumaric acid	2.53 ± 0.23	16.2 ± 1.46
Vanillic acid	<4.90 ^b^	<31.4
Gallic acid	7.11 ± 0.64	45.5 ± 4.09
Caffeic acid	4.73 ± 0.33	30.3 ± 2.12
5–*O*–caffeoylquinic acid (chlorogenic acid)	1501 ± 75.07	9610 ± 480
Flavonoids		
Catechin	28.1 ± 2.81	180 ± 18.0
Kaempferol–3–*O*–glucoside	11.3 ± 0.45	72.1 ± 2.88
Luteolin–7–*O*–glucoside	<2.45	<15.7
Quercetin	<19.6	<125
Quercetin–3–*O*–glucoside + Quercetin–3–*O*–galactoside	31.4 ± 1.88	201 ± 12.0
Rutin	9.80 ± 0.29	62.7 ± 1.88
Quercitrin	1.37 ± 0.08	8.77 ± 0.53
Chrysoeriol	<0.30	<1.92
Naringenin	3.94 ± 0.26	25.2 ± 1.77
Isorhamnetin	<4.90	<31.4
Coumarins		
Scopoletin	<1.20	<7.68
Total phenolics ^c^	4.74 mg/g de	30.3 mg/L SJPW

^a^ Results are given as content (µg/g dry extract and µg/L SJPW) expressed as mean ± measurement error of the method (obtained in method validation), ^b^ concentration is below limit of quantification (LOQ), ^c^ Sum of the contents of all quantified compounds by LC–MS/MS.

**Table 3:** Total phenolic and flavonoid contents and antioxidant activity of SJPW.

Parameter (Unit)	Value	
Total phenolics	8.52 ± 0.37 * mg GAE/g de	54.5 ± 2.37 mg GAE/L SJPW
Total flavonoids	0.48 ± 0.03 mg QE/g de	3.04 ± 0.17 mg QE/L SJPW
DPPH^•^ IC_50_	0.69 ± 0.03 mg/mL	/
FRAP	4.43 ± 0.28 mg AAE/g de	28.4 ± 1.79 mg AAE/L SJPW

* values are means ± SD of three measurements, de—dry extract, GAE—gallic acid equivalents, QE—quercetin equivalents, AAE—ascorbic acid equivalents, SJPW—strawberry juice production wastewater.

## Data Availability

The original contributions presented in the study are included in the article, and further inquiries can be directed to the corresponding author.
